# Coronavirus Disease 2019 (COVID-19) Accompanied by Maculopapular Rash: A Case Study

**DOI:** 10.7759/cureus.10414

**Published:** 2020-09-12

**Authors:** Eda Öksüm Solak, Burcu Baran Ketencioğlu, Salih Levent Çinar, Demet Kartal, Murat Borlu

**Affiliations:** 1 Dermatology and Venereology, Erciyes Universty, Kayseri, TUR; 2 Respiratory Medicine, Erciyes University, School of Medicine, Kayseri, TUR; 3 Dermatology, Erciyes University, Kayseri, TUR

**Keywords:** drug rash, sars-cov-2

## Abstract

A new type of coronavirus (coronavirus disease 2019; COVID-19), which emerged in the People's Republic of China, spread all over the world over time and became a pandemic. Dermatological symptoms seen during the course of the disease have gained importance over time. Studies have shown that many dermatological findings such as erythematous rash, urticaria, pseudo-chilblain, maculopapular, livedo/necrosis, and vesicular lesions may accompany the disease. In this study, a 24-year-old female patient with maculopapular lesions who had no previous history of allergy or dermatological disease and regressed without any dermatological treatment is presented.

## Introduction

An infection caused by a new type of coronavirus (coronavirus disease 2019; COVID-19) emerged in December 2019 in the Hubei province of the People’s Republic of China. It had spread across the world by March 11, 2020, and was accepted as a pandemic [1,2]. Although the first symptom of COVID-19 was pneumonia, symptoms such as cough, fever, nasal congestion, fatigue, gastrointestinal symptoms, and others of an upper respiratory tract infection were also seen in the course of the disease [1]. Dermatological symptoms seen during the course of the disease have also gained importance over time. This study aimed to present the case of a patient with COVID-19 accompanied by a maculopapular rash.

## Case presentation

**Figure 1:**
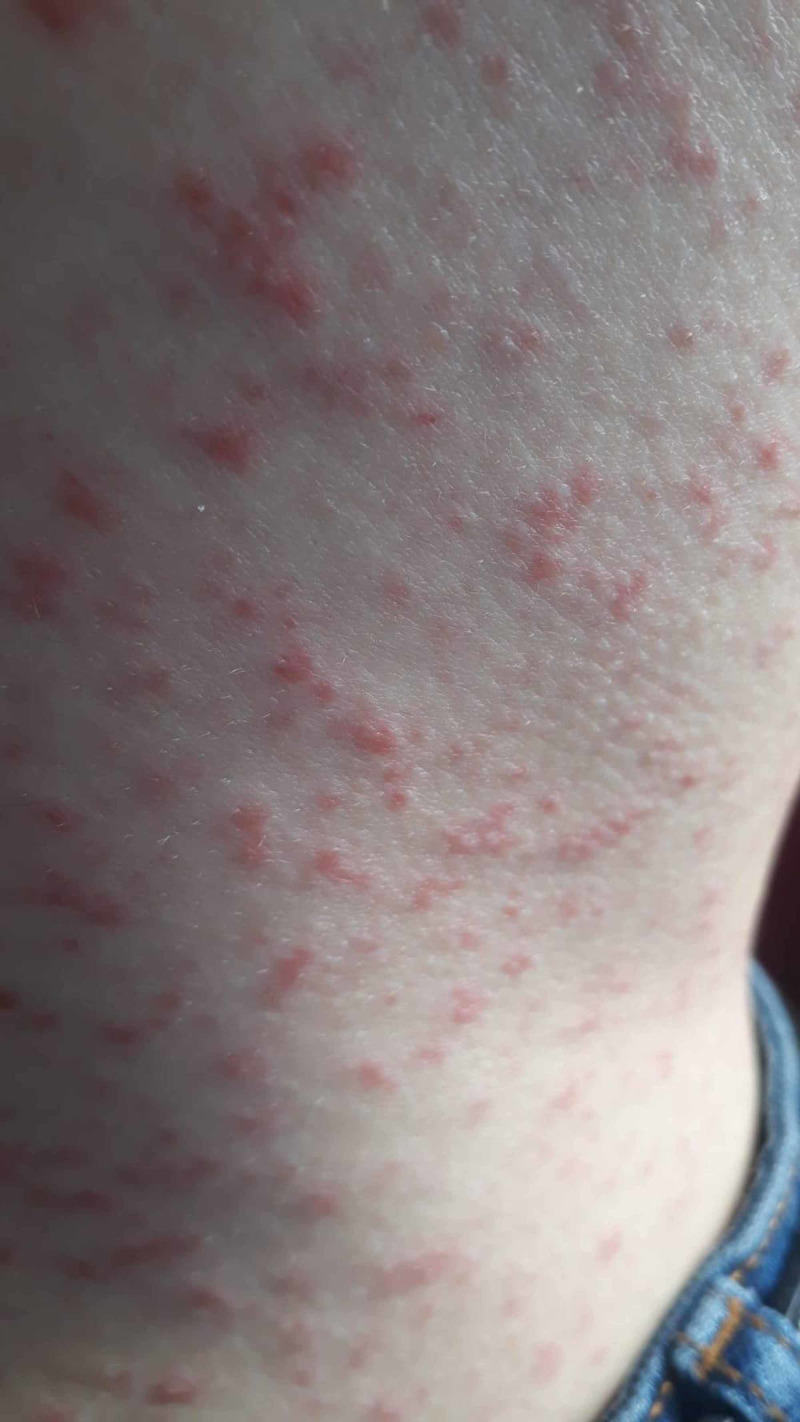
Erythematous rash on the body

**Figure 2:**
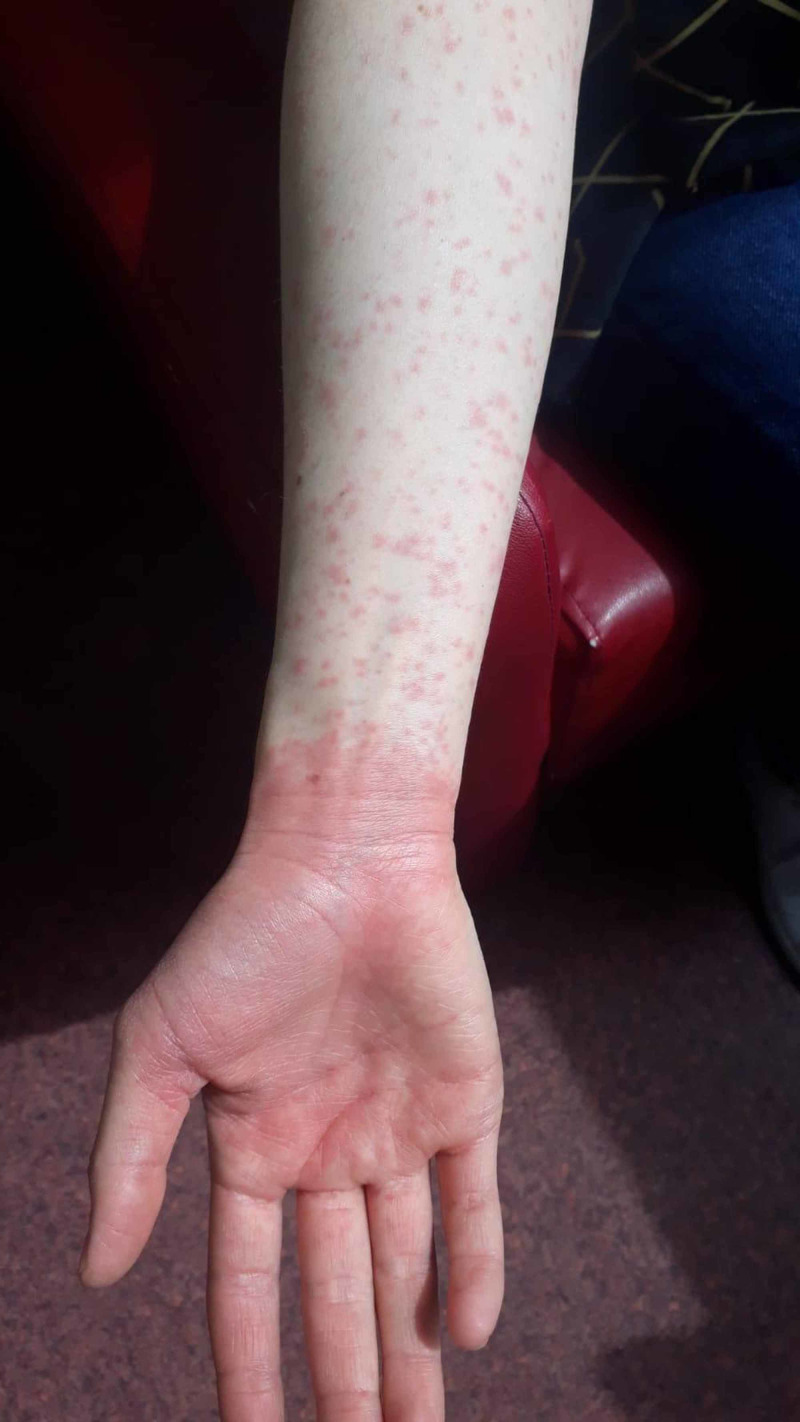
Erythema in the right palm

A 24-year-old female patient was admitted to the emergency clinic due to a widespread erythematous rash on the face and body. She was later transferred to the emergency respiratory infection outpatient clinic, specially created for COVID-19, because she also had a fever and cough. On being evaluated in the COVID-19 clinic, she was found to have widespread erythematous maculopapular lesions throughout the body, which were more prominent on the arms and legs (Figure 1). The lesions were 1-2 mm in diameter, scattered on the body, and separated from each other, while some formed patches and plaques. Some of the lesions were petechial, which did not fade after being pressed. Significant erythema was noted in the right palm (Figure 2). The oral and conjunctival mucosae were normal.

**Figure 3:**
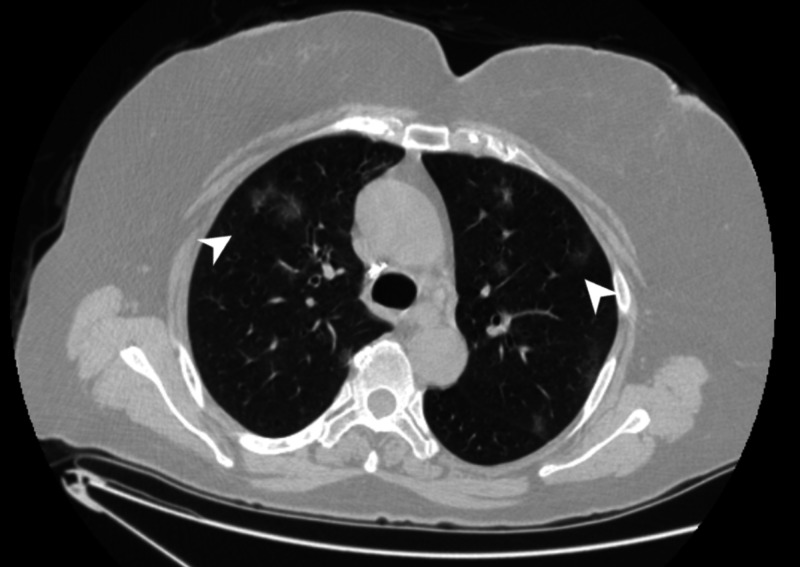
Peripheral ground-glass appearance in both lungs

The anamnesis revealed that the patient visited the outpatient clinic nine days ago due to a cough and fever, which lasted now for about 10 days. She was diagnosed with atypical pneumonia, after which gemifloxacin treatment was started. On the seventh day of medical treatment, the rash began to form on her body. The patient did not have any additional disease. Although she had used the same medication two times earlier for other complaints, she never experienced any side effects. The clinical lesions of the patient were interpreted as maculopapular drug eruption. The patient suffered from this condition for 10 days before coming to the clinic, which was the 16th day of the first coronavirus case detected in Turkey. She also had a history of foreign travel, was in close contact with a traveler, and had a fever. Thus, she was evaluated for the possibility of COVID-19. As the patient's cough did not regress and she continued to exhibit a fever, a polymerase chain reaction test with a mucosal swab on suspicion of COVID-19 was conducted, but the result was negative. However, the chest computed tomography (CT) showed pneumonia compatible with COVID-19. Based on the symptoms and CT imaging, the patient was diagnosed with COVID-19 (Figure 3) [3,4]. There was minimal lymphopenia, eosinophil, serum reactive protein (CRP), alanine transferase (ALT) and aspartate transaminase (AST) elevation in the blood tests of the patient. Immunoglobulin G and M checked for measles were negative. The patient was administered hydroxychloroquine, oseltamivir, and azithromycin treatment due to the diagnosis of COVID-19. The pneumonia complaint significantly improved on the fifth day. Only emollient cream was recommended for the skin rashes of the patient. In parallel with the improvement in pneumonia, the skin lesions regressed without any additional treatment. The patient was discharged with strict instructions that she complied with the quarantine conditions. During the check-up, the patient did not exhibit any lung or skin complaints or findings.

## Discussion

In the first publication on skin findings of COVID-19 infection, patients who used medication 15 days before admission were excluded, and erythematous rash, urticaria, and varicelliform eruption were observed in patients [5]. Galvan et al. classified dermatological findings of COVID-19 as pseudo-chilblain, urticarial, maculopapular, livedo/necrosis, and vesicular lesions at theır study [6]. In another study, as a result of the survey applied to 382 patients diagnosed with COVID-19, it was stated that at least one of the complaints of rash, redness, bruising and wound was found in 70 (18.3%) patients [7].

Exanthematous drug reactions occur in 1-5% following the first use of many drugs and usually emerge between the fourth and the 21st day of drug treatment [8]. In the present case, the patient did not have any history of allergic reactions due to the use of gemifloxacin or any other drug. The patient was given topical moisturizers as a treatment and was followed up. As a result of the treatment and recovery from COVID-19, the skin lesions also regressed dramatically, which was unexpected for a drug eruption. Viral infections have been shown to increase the incidence of drug eruptions and make them more severe, long-lasting, and resistant. Although the skin rash in the present case was an exanthematous drug rash, COVID-19 played a significant role in its formation. The reasons were the typical lung complaints of the patient, the presence of the pandemic in Turkey, the fact that the patient had used the same drug twice earlier with no rash, and the fact that the rash was severe and widespread. This was also because of the fact that the clinical symptoms responded very well to COVID-19 treatment and that the skin lesions recovered quickly without the need for additional treatment.

## Conclusions

Although no previous allergic history has been found in patients with COVID-19 infection, allergic reactions should be considered in COVID-19 infection. The disease should be carefully evaluated after implementing necessary measures, and a COVID-19 test should be performed if a drug eruption is observed in a severe, widespread, and pandemic region.
